# Supplementary material for: Seminoma and Embryonal Carcinoma Footprints Identified by Analysis of Integrated Genome-Wide Epigenetic and Expression Profiles of Germ Cell Cancer Cell Lines
Source: PLoS One. 2014 Jun 2;9(6):e98330. doi: 10.1371/journal.pone.0098330 (PMC4041891; doi:10.1371/journal.pone.0098330)
Supplement: File S1 — ZIP file containing DMRforPairs output for significant regions. Please start from the html files. (ZIP) [file pone.0098330.s008.zip › figures/37214.pdf]

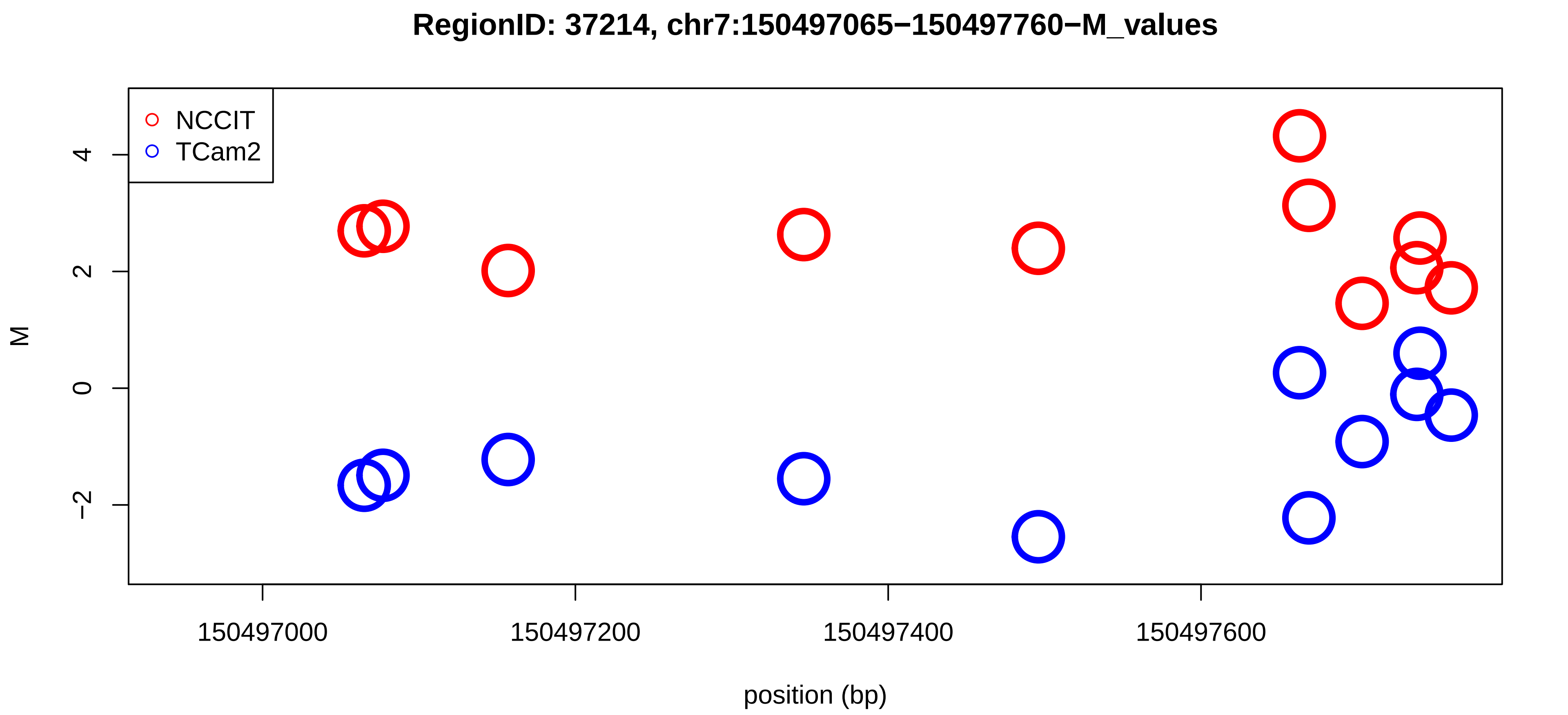
RegionID: 37214, chr7:150497065−150497760−M_values
NCCIT
TCam2
M
4
2
0
−2
150497000
150497200
150497400
150497600
position (bp)

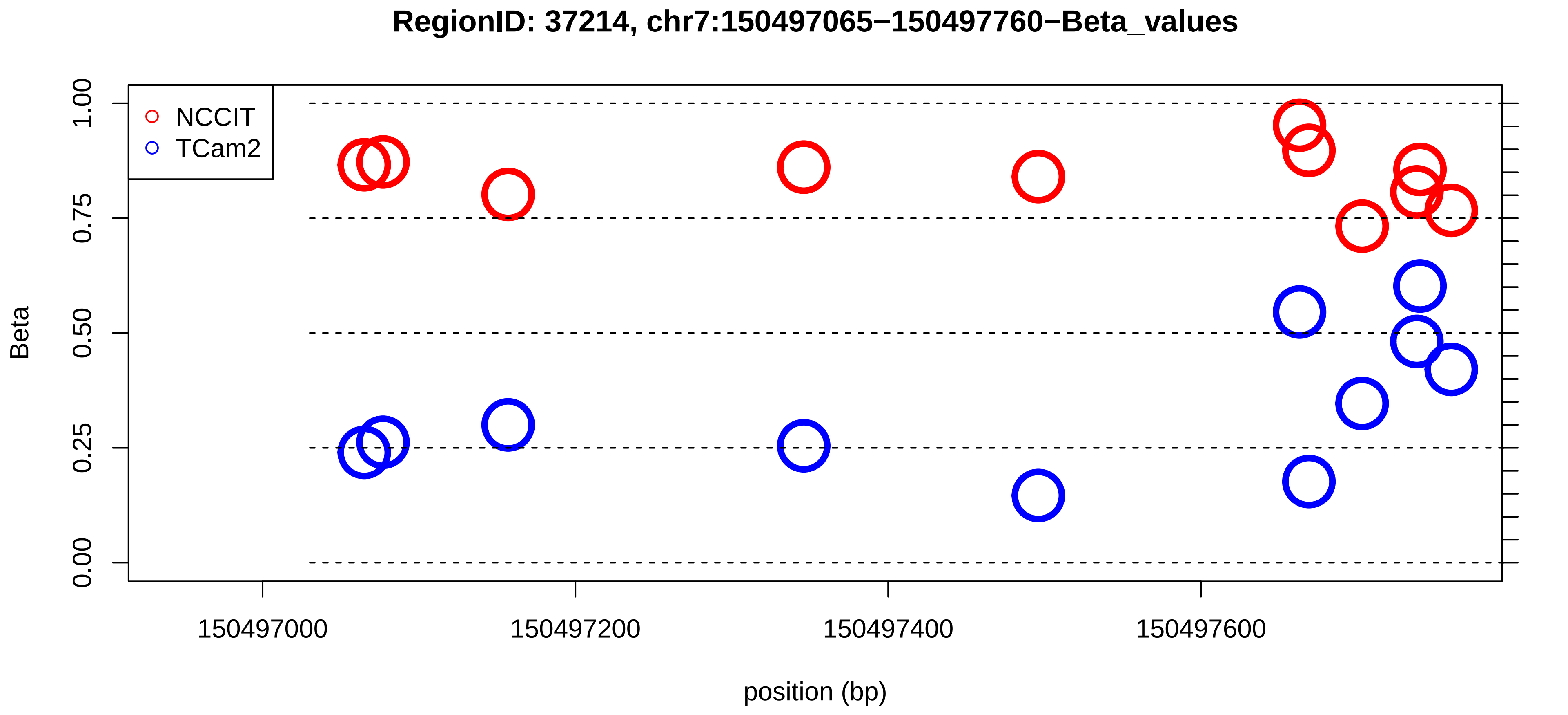
RegionID: 37214, chr7:150497065−150497760−Beta_values
NCCIT
TCam2
Beta
1.00
0.75
0.50
0.25
0.00
150497000
150497200
150497400
150497600
position (bp)

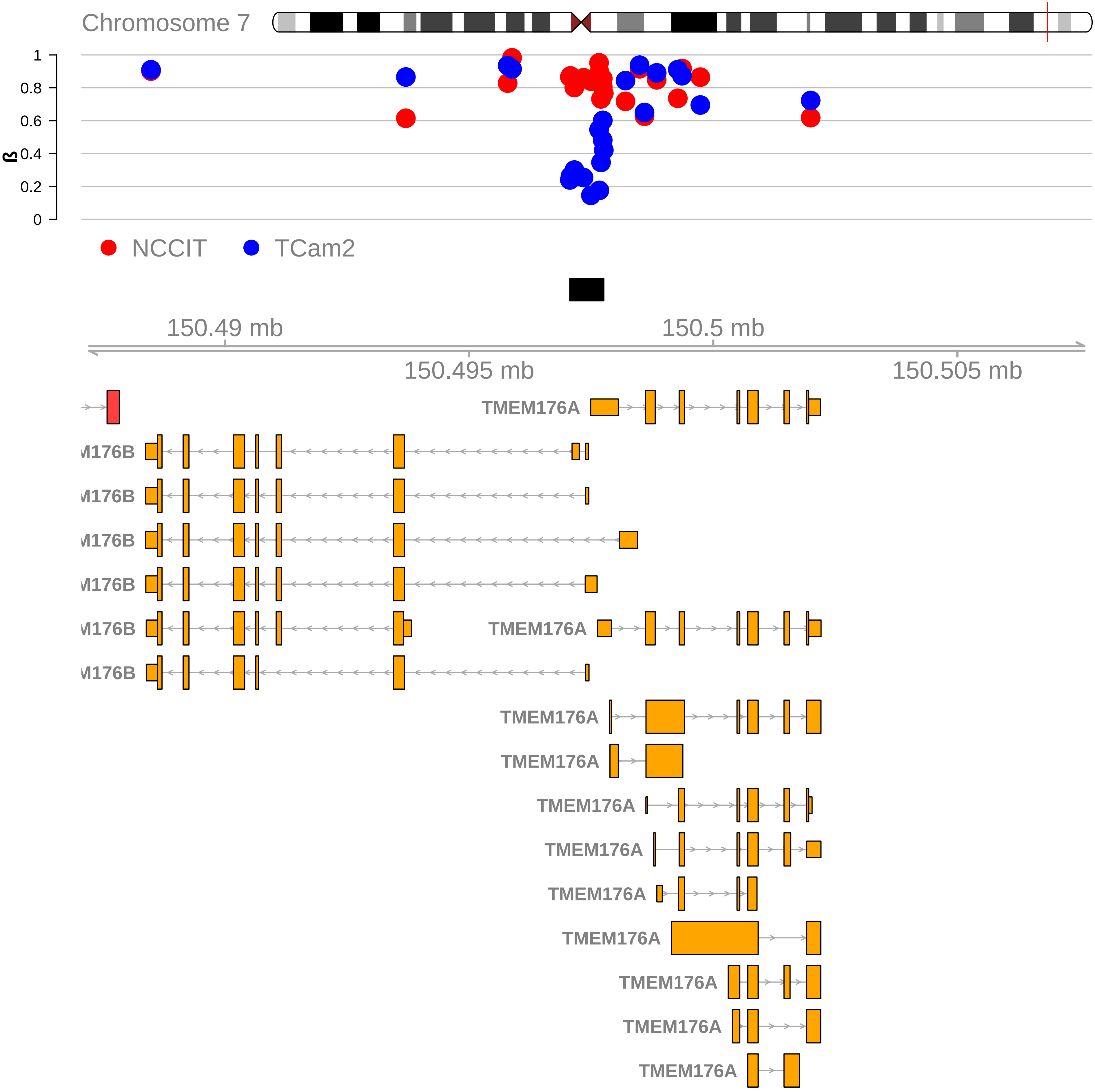

Chromosome 7
ß
1
0.8
0.6
0.4
0.2
0
NCCIT
TCam2
150.49 mb
150.5 mb
150.495 mb
150.505 mb
TMEM176A
M176B
TMEM176A
